# Gambetta’s Brain Examined

**Published:** 1886-06

**Authors:** 


### GAMBETTA'S BRAIN EXAMINED.

Conformably with the practice in vogue of investigating the peculiarities of form and structure presented by the brains of famous men, that of M. Gambetta has been duly examined by MM. Duval and Chudinski. The report of these experts was read at a meeting of the Anthropological Society of Paris. We await details of weight and other essential particulars, but certain facts already brought to light regarding Gambetta's cerebrum verify in a remarkable fashion some of the latest deductions of physiology regarding the functions of the brain. We find the observers recounting, first, the high development of the speech center in the third left frontal region. Nor was this portion of the statesman's brain found to be merely well developed. It actually exhibited a double folding or reduplication in this area, indicating an exceptionally active disposition as far as eloquence and command of language were concerned—qualities for which Gambetta, of all men, was markedly distinguished. The Parisian savants tell us that in the brains of Wutfert the lawyer, and Huber the philosopher, both remarkable for their rhetorical ability, the convolution already noted was singularly developed, and was more wavy and more complex than in ordinary brains. In these cases, however, there was no double fold, as in the brain of Gambetta, which in other respects showed certain peculiarities of development. In the forehead region—admittedly the seat of the highest intellectual powers—the statesman's brain showed complexity of folding associated with great diagramatic regularity. Altogether, the examination in question is of a highly interesting character, proving, as it does, the fact that evidences of genius and ability are not left unrecorded on the organ of mind. How far training and education may modify brain structure is as yet a moot-point of science. One thing, however, is certain—namely, that it is quality and not quantity of brain matter which primarily dominates the world. That a large brain may be associated with low intellectual powers is a proved fact. Given a large brain mass and a high quality of structure, derived from an educated ancestry or from a sound stock in other respects, and we find represented the conditions which subdue all things to the will of the individual, which control the destinies of nations, and which revolutionize the world of letters, art or science.

